# Clinical nurses as digital guardians: unlocking the key determinants of digital resilience in the AI era

**DOI:** 10.3389/fpubh.2026.1794227

**Published:** 2026-06-19

**Authors:** Ming Yu, Jiyang Chen, Rong Yu, Mengjia Zhou, Xiaoli Fan, Ronghui Geng, Lingling Jiang

**Affiliations:** 1 Department of Nursing, Affiliated Rudong Hospital of Xinglin College, Nantong University, Nantong, Jiangsu, China; 2 Jiangsu Vocational College of Business, Nantong, Jiangsu, China

**Keywords:** AI literacy, clinical nurses, digital resilience, organizational support, self-efficacy

## Abstract

**Background:**

As artificial intelligence (AI) integrates into healthcare, clinical nurses need digital resilience to adapt to technological changes. However, little is known about the digital resilience of practicing nurses. This study aimed to investigate the current status and influencing factors of digital resilience among clinical nurses.

**Design:**

A quantitative, descriptive, cross-sectional study was conducted in two tertiary hospitals in Nantong City, China.

**Methods:**

A convenience sample of 460 clinical nurses completed questionnaires assessing general characteristics, AI literacy, organizational support, self-efficacy, and digital resilience. Multiple linear regression was used to identify influencing factors.

**Results:**

The mean digital resilience score was 123.05 ± 11.80 (possible range 39–195), indicating a moderate-to-high level. Monthly income, number of night shifts per month, participation in AI-related training, AI literacy, organizational support, and self-efficacy were significant predictors (all *P* < 0.05). These variables collectively accounted for 78.9% of the variance.

**Conclusion:**

Digital resilience among clinical nurses is influenced by multiple factors. Nursing managers should implement systematic training, optimize shift schedules, enhance organizational support, and foster self-efficacy to improve digital resilience.

## Introduction

Against the backdrop of global digital transformation in healthcare, the in-depth integration of artificial intelligence (AI) and healthcare brings both opportunities and challenges to nursing practice (1). As frontline implementers of technological innovation, clinical nurses act as a bridge between clinical nursing and digital technology. They also serve as core “digital guardians” in a technology-driven medical environment. They shoulder the important mission of safeguarding patient safety, data security and the humanistic essence of nursing (2). This dual role highlights the core value of digital resilience. With the advancement of China's “Digital China” strategy, AI and other digital technologies have been widely applied in clinical nursing. Nurses' digital resilience is directly related to the quality of nursing services and the effectiveness of digital transformation.

Digital resilience refers to the ability to adapt, learn and make continuous adjustments in the process of digital transformation. It is a key psychological resource to cope with digital changes. It is also an important factor affecting the effectiveness of technology application and the quality of nursing care (3). Mo et al. (4) used the Walker & Avant concept analysis method to systematically review literature from multiple databases. They clarified the four core attributes of clinical nurses‘ digital resilience and confirmed its key impact on nursing quality. Many studies have shown that employees with higher digital resilience can better cope with the impact of technological changes (4). They take the initiative to learn digital skills and adopt positive coping behaviors. This in turn promotes their professional growth (5). A cross-sectional quantitative study involving 237 registered nurses found that the rational application of information and communication technology (ICT) tools can effectively enhance nurses' resilience, and nurses with higher resilience showed better job adaptability and professional development potential (6).

Current research on nurses‘ digital resilience reveals several important gaps. The research focus has been biased: most existing studies concentrate on technology itself or on nurses' overall psychological resilience, rather than on digital resilience specifically (6–8). In terms of study populations, most surveys have targeted nursing students (9), with only a few investigations providing an initial definition of digital resilience among clinical nurses (4). Yet clinical practicing nurses are the frontline practitioners of digital healthcare, and systematic empirical research on their digital resilience remains lacking (4, 10). This gap cannot adequately support the needs of clinical practice. Furthermore, the depth of existing research is insufficient. A recent review noted that most studies are limited to descriptive status reports or single-factor analyses (8), and they lack systematic exploration of the mechanisms that influence digital resilience (11). Consequently, it is difficult to clarify the synergistic relationships among various contributing factors, and precise guidance for clinical interventions is still unavailable (12).

Based on the above research background, this study adopts the multi-dimensional “Technology-Individual-Organization (TIO)” framework as its theoretical guide. This framework is derived from the Technology-Organization-Environment (TOE) framework originally proposed by Tornatzky and Fleischer in 1990 (13). The TOE framework posits that technology adoption is influenced by three contextual factors: technological, organizational, and environmental. This framework has been widely applied across healthcare settings (14). Scholars have since optimized and adapted this framework for application in the medical field, refining it to emphasize the synergistic effect of three dimensions. The technical dimension includes the characteristics and application scenarios of digital technology. The individual dimension covers nurses‘ digital literacy, self-efficacy, and other related factors. The organizational dimension refers to the training and support systems provided by medical institutions. This adapted TIO framework has been widely used in research related to the adoption of digital medical technology and the digital adaptation of healthcare workers. For instance, Lin employed a technology-individual fit perspective integrated with organizational readiness to investigate the effectiveness of mobile nursing information systems, demonstrating that technology-individual fit is the most influential factor in system usage among clinical nurses (15). More recently, Zeng et al. applied a conceptually aligned multi-dimensional lens in their study of 901 nurses across three Chinese provinces, confirming that AI literacy promotes nurses' AI usage intention both directly and indirectly via self-efficacy and general attitudes, thereby providing robust empirical support for the TIO framework in the context of AI adoption in nursing (16).

Building on this TIO framework, digital resilience is shaped by individual capabilities, internal beliefs, and the organizational environment (4). Within this framework, AI literacy serves as the foundation for nurses to understand and apply AI technology (17). Zeng et al. (16) further confirmed this mechanism in a multi-center survey of 901 nurses. Self-efficacy enables nurses to actively respond to technological challenges and overcome digital adaptation difficulties. Among 1,081 Italian nurses and students, only 47.1% had adequate digital self-efficacy; female nurses had twice the odds of inadequacy (OR = 2.038), and each additional year of work experience reduced odds by 4% (OR = 0.955) (18). Organizational support provides a key external guarantee (19). Chang et al. (6) surveyed 237 nurses and highlighted the necessity of organizational initiatives to promote effective use of digital tools. Although existing studies have identified these influencing factors, the synergistic mechanism has not been fully empirically verified, nor supported by a mature theoretical framework. These deficiencies highlight the core value of this study.

This study aims to clarify clinical nurses‘ digital resilience and analyze how AI literacy, self-efficacy, and organizational support influence it, filling current research gaps.

The study has theoretical and practical value. Theoretically, it extends the digital resilience framework (4) and provides a TIO-based foundation for future research. Practically, it identifies key factors and offers evidence to optimize digital training and support systems (20). Ultimately, this will enhance nurses' digital adaptation, reduce burnout, and advance smart nursing (21).

Based on the TIO framework and existing evidence, this study puts forward the following hypotheses:

H1: Higher AI literacy is positively correlated with the digital resilience of clinical nurses.
H2: Higher self-efficacy is positively correlated with digital resilience.
H3: The higher the perceived organizational support, the more positive its correlation with digital resilience.
H4: Demographic factors and occupational factors (monthly income, night shift frequency and AI-related training) are also associated with digital resilience.

## Methods

### Study design

This is a quantitative, descriptive, cross-sectional study.

### Participants and settings

This quantitative, cross-sectional study was conducted in two tertiary hospitals in Nantong City, Jiangsu Province, China. The target population comprised all registered clinical nurses working in these two hospitals between August and September 2025. The total number of eligible nurses was 780 (Hospital A: 410, Hospital B: 370). A convenience sampling method was used to recruit participants (22). All eligible nurses were invited to participate voluntarily, and 460 completed the survey, with a response rate of 58.97%.

Inclusion criteria were: (1) being a registered clinical nurse; (2) having at least 6 months of clinical nursing experience; (3) being aged 18 years or older (the legal working age in China); and (4) providing informed consent. Exclusion criteria were: (1) nurses undergoing further training or internships; (2) non-clinical staff (e.g., nursing department administrators); (3) nurses on extended leave.

The required sample size was calculated using the formula for multiple linear regression: (*N* = 10^*^k + 50), where *k* is the number of independent variables (23). Our final regression model included 11 independent variables (age, education, service length, title, income, night shifts, overtime, AI training, AI literacy, organizational support, self-efficacy). Thus, the minimum sample size was (10 ^*^11 + 50 = 160). Accounting for a 20% invalid response rate, we aimed for at least 200 participants. Ultimately, 460 clinical nurses completed the survey, exceeding the requirement.

### Theoretical framework and questionnaire constructs

## Theoretical framework

**Figure 1:**
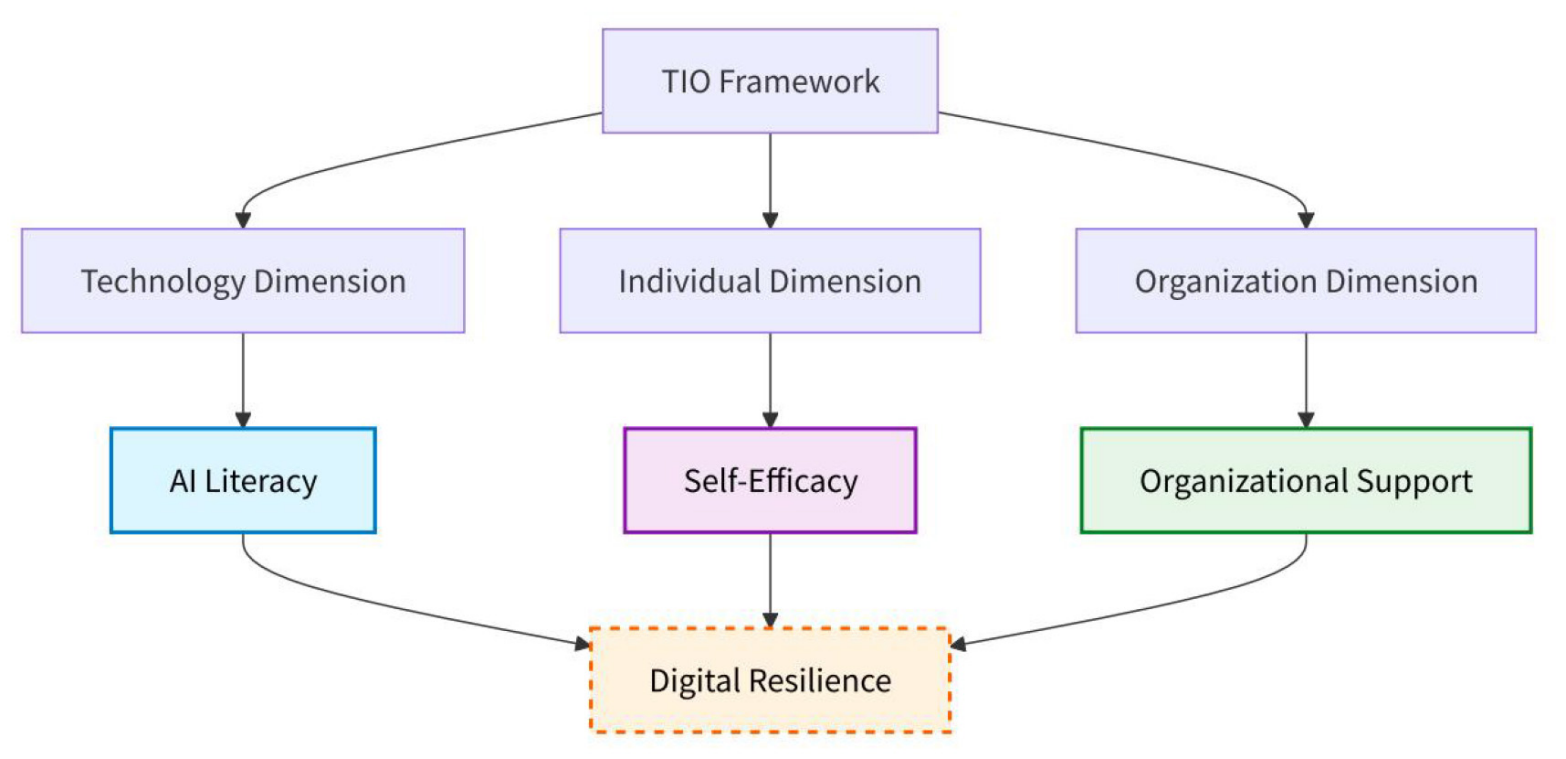
Conceptual diagram of the Technology-Individual-Organization (TIO) framework. The diagram illustrates the three core dimensions of the TIO framework and their hypothetical relationships with digital resilience. AI literacy (technology dimension), self-efficacy (individual dimension), and organizational support (organization dimension) are the key influencing factors, collectively affecting the outcome variable (digital resilience).

This study was guided by a multi-dimensional “technology–individual–organization” framework. Accordingly, the questionnaires were selected to capture three core constructs: Technology-related construct: AI literacy, measured by the Artificial Intelligence Literacy Scale (AILS) (24). Individual-related construct: Self-efficacy, measured by the General Self-Efficacy Scale (16). Organization-related construct: Perceived organizational support, measured by the Nurses' Organizational Support Scale (25). Outcome construct: Digital resilience, measured by the Digital Resilience Assessment Scale for Nursing Undergraduates (26). The general information questionnaire captured demographic and occupational characteristics (age, gender, education, years of service, title, income, night shifts, AI training participation). All constructs were operationalized according to the original scale developers' definitions (Figure 1).

## General information questionnaire

A self-designed general information questionnaire was used to collect demographic and occupational characteristics of clinical nurses. The collected data included age, gender, educational background, years of service, professional title, monthly income, frequency of night shifts per month, and participation in AI-related training.

## Artificial intelligence literacy scale, AILS

This scale, developed by Wang et al., uses a 7-point Likert scale (from “strongly disagree” to “strongly agree”) (24). The 7-point format was retained from the original scale validation study, as it provides greater granularity in capturing nuanced attitudes toward AI literacy compared to a 5-point scale, which may reduce central tendency bias and improve discriminative power (24). In a survey of Turkish surgical nurses, the Cronbach's α coefficient for this scale was 0.821 (27). In this study, the Cronbach's α coefficient for this scale was 0.858.

## Nurse organization support scale

The Organizational Support Scale was developed by Chen Zhixia in 2006 and revised by Zuo Hongmei et al. in 2009, forming the Nurses' Organizational Support Scale (28). The 5-point Likert format (1 = strongly disagree to 5 = strongly agree) was retained from the original validation study, as it has been widely applied and validated in Chinese nursing populations. The scale comprises two dimensions: affective support (Items 1–10) and instrumental support (Items 11–13), totaling 13 items. The total score ranges from 13 to 65, with higher scores indicating a stronger sense of organizational support. The level of organizational support is determined by the average score of all items: 1.00–2.33 = low support, 2.34–3.66 = moderate support, and 3.67–5.00 = high support. In this study, the Cronbach's α coefficient was 0.980.

## The general self-efficacy scale

The General Self-Efficacy Scale is a unidimensional scale developed by Schwarzer et al. and adapted into Chinese by Wang Caikang et al. in 2001 (29). The 4-point Likert format (1 = completely incorrect to 4 = completely correct) follows the Chinese adaptation, which has demonstrated good reliability and validity in Chinese populations (5). The scale consists of 10 items, with total scores ranging from 10 to 40. Higher scores indicate better self-efficacy among nurses. In this study, the Cronbach's α coefficient was 0.871.

## Digital resilience assessment scale for nursing undergraduates

Developed by Li Fanfan, this self-administered scale comprises 39 items across five dimensions: perceived digital risk (11 items), cognitive coping strategies (6 items), knowledge and skill acquisition (10 items), overcoming digital stress (5 items), and adaptation to digital environments (7 items) (30). The 5-point Likert format (1 = completely disagree to 5 = completely agree) was developed and validated specifically for nursing undergraduates by Li Fanfan (2025) and demonstrated high internal consistency (Cronbach's α = 0.958) in the original validation study. The total score ranges from 39 to 195, where higher scores reflect greater digital resilience. In this study, the Cronbach's α coefficient was 0.958.

## Data collection

Data were collected via electronic questionnaires distributed through WeChat. A QR code linking to the questionnaire was sent to nursing staff in clinical departments. The preface of the questionnaire clearly stated the study purpose, completion instructions, and privacy protection measures. Participation was voluntary. Informed consent was obtained from all participants before they started the questionnaire.

To ensure data completeness, the questionnaire had a mandatory response feature. This feature prevented submission until all items were answered. It also provided real-time reminders for any unanswered questions. An anonymous survey mechanism was implemented. Technical measures were used to restrict each user to a single submission. This eliminated duplicate responses.

Invalid questionnaires were excluded. They were defined as those with a completion time of less than 120 s or those showing patterned responses. A total of 460 questionnaires were returned.

## Pilot testing and content validity

Before the main survey, the questionnaire battery was piloted with 30 clinical nurses who met the inclusion criteria (not included in the final sample). Pilot testing is recommended to identify potential issues with item clarity, length, and relevance before large-scale administration (31). Feedback was collected on item clarity, length, and relevance. Minor wording adjustments were made for clarity. Content validity was assessed by a panel of five experts (two nursing professors, two clinical nurse managers, and one health informatics specialist). The item-level content validity index (I-CVI) ranged from 0.80 to 1.00, and the scale-level CVI (S-CVI/Ave) was 0.94, indicating good content validity (32).

## Statistical analysis

Data analysis was performed using SPSS 26.0 software. Descriptive statistical analysis was conducted for all variables. Categorical data were presented as frequency and percentage. Normally distributed continuous variables were expressed as mean ± standard deviation, while non-normally distributed data were presented as median and interquartile range.

Independent samples *t*-test and one-way ANOVA were employed to compare digital resilience scores among groups with different demographic characteristics. Univariate analysis identified statistically significant variables (*P* < 0.05) for inclusion in multiple linear regression analysis as independent variables to determine factors influencing digital resilience. Cronbach's α coefficient was used to assess scale reliability, with values above 0.70 considered acceptable (33). All statistical tests were performed at a significance level of *P* < 0.05.

## Results

### Sociodemographic characteristics of participants

Among the 460 clinical nurses, 458 (99.6%) were female and 2 (0.4%) were male. The mean age was 34.2 ± 8.1 years, with the largest age group being 31–40 years (199, 43.3%). Regarding educational background, 139 (30.2%) held a vocational secondary school degree, 234 (50.9%) a junior college degree, and 87 (18.9%) a bachelor's degree or higher. A total of 192 (41.7%) held a senior nurse practitioner title or above. The majority (402, 87.4%) had not participated in any AI-related training.

## Scores of clinical nurses' artificial intelligence literacy scale, organizational support scale, self-efficacy, and digital resilience

According to the scoring protocol of the Digital Resilience Scale, a total score of 39–91 indicates low resilience, 92–143 indicates moderate resilience, and 144–195 indicates high resilience. Thus, the mean score of 123.05 ± 11.80 falls within the moderate range, leaning toward the upper level. For the AI Literacy Scale (possible range 12–84), a score of 50.23 ± 4.84 indicates a moderate level. The Organizational Support Scale (possible range 13–65) yielded a mean of 50.11 ± 4.29, corresponding to a high level of perceived support (average item score 3.67). The Self-Efficacy Scale (possible range 10–40) gave a mean of 31.04 ± 1.55, indicating a relatively high level. Table 1 presents the scores of each scale and subscale. Within each scale, dimensions are sorted in descending order of mean scores to highlight areas of relative strength and weakness.

**Table 1:** Scores of digital resilience, perceived organizational support, self-efficacy, and AI literacy among clinical nurses (*n* = 460).

Project	Minimum value	Maximum value	Overall average score	Sorting
Consciousness	9	17	13.04 ± 1.4	1
Using	9	15.5	12.11 ± 1.16	4
Evaluation	9	16.1	12.5 ± 1.2	3
Ethics	9	16.2	12.59 ± 1.19	2
AI literacy	36	64.8	50.23 ± 4.84	
Perceiving digital risks	26	42	32.34 ± 3.16	1
Cognitive coping strategies	16	24	19.66 ± 1.84	4
Acquiring knowledge and skills	26	40	31.9 ± 3.12	2
Overcoming digital stress	13	22	16.67 ±−1.65	5
Adapting to the digital environment	18	28	23 ±−2.27	3
Digital resilience	99	151	123.05 ±−11.8	
Emotional support	30	44	37.28 ±−3.42	1
Instrumental support	10	15	12.84 ±−1.2	2
Perceived organizational support	40	59	50.11 ±−4.29	–
Self-efficacy	25	39	31.04 ±−1.55	–

## Comparison of total digital resilience scores among nurses with different characteristics (Table 2)

Table 2 presents the comparison of digital resilience scores across different demographic and occupational characteristics. Significant differences were observed for age, educational background, length of service, professional title, monthly income, number of night shifts, overtime work, and AI-related training participation (all *P* < 0.05).

**Table 2:** Comparison of digital resilience scores among clinical nurses (*n* = 460,$\overset{¯}{X}\pm S\;$).

Project		*N*	Score	t/F	*P*
Age (years)	≤ 25	70	117.54 ± 9.84	19.162	*** < 0.001***
	26–30	126	119.06 ± 7.79		
	31–40	199	126.17 ± 12.7		
	40	65	127.14 ± 12.86		
Educational background	Vocational secondary school	139	119.2 ± 9.87	136.179	*** < 0.001***
	Junior college	234	119.79 ± 9.05		
	Bachelor's degree or higher	87	137.95 ± 9.37		
Length of service (years)	≤ 3	137	119.34 ± 9.5	29.379	*** < 0.001***
	4 ~ 6	82	119.56 ± 7.57		
	7 ~ 10	70	117.69 ± 9.65		
	11 ~ 15	69	128.45 ± 12.08		
	≥15	102	130.85 ± 13.18		
Professional Title	Nurse and below	253	118.32 ± 8.77	−9.408	*** < 0.001***
	Senior Nurse Practitioner and above	192	127.98 ± 12		
Monthly income(RMB)	≤ 4,000	79	115.32 ± 7.97	62.98	*** < 0.001***
	4,001 ~ 6,000	150	119.27 ± 9.05		
	6,001 ~ 8,000	72	120.65 ± 8.98		
	≥8,000	159	131.53 ± 11.89		
Number of night shifts per month	0	105	131.84 ± 12.14	29.944	*** < 0.001***
	1 ~ 3	44	120.75 ± 9.78		
	4 ~ 9	291	120.41 ± 10.62		
	≥10	20	120.3 ± 8.25		
Whether working overtime (at least once per month in the past 12 months)	Have	436	122.62 ± 11.55	−3.357	***0.001***
	None	24	130.83 ± 13.79		
Whether to participate in AI-related knowledge training	Have	58	134.09 ± 7.6	10.993	*** < 0.001***
	None	402	121.45 + −11.45		

Bold values indicate statistical significance (*P* < 0.05).

## Multifactorial analysis of clinical nurses' digital resilience

Using the total score of clinical nurses' digital resilience as the dependent variable, multivariate linear regression analysis was conducted with variables showing statistical significance in univariate analysis as independent variables.

The results indicated that monthly income (β = 1.093, *P* = 0.001), number of night shifts per month (β = −1.242, *P* = 0.001), participation in AI-related training (β = −2.410, *P* = 0.010), AI literacy (β = 0.523, *P* < 0.001), perceived organizational support (β = 1.729, *P* < 0.001), and self-efficacy (β = 0.527, *P* = 0.002) were significant influencing factors. These factors collectively explained 78.9% of the total variance in Clinical Nurses' Digital Resilience. The regression model was statistically significant (*F* = 156.816, *P* < 0.001). (Table 3).

**Table 3:** Multiple linear regression analysis of influencing factors of digital resilience among clinical nurses (*N* = 460).

Project	β	Standard error	*β'*	*t*	*p*
(Constant)	−0.894	7.441		−0.12	0.904
Age	−0.701	0.484	−0.054	−1.449	0.148
Educational background	0.749	0.542	0.044	1.382	0.168
Length of service (years)	−0.229	0.305	−0.03	−0.751	0.453
Professional title	0.241	0.788	0.011	0.305	0.76
Monthly income (RMB)	1.093	0.327	0.104	3.342	***P** **=** **0.001** **<** **0.05***
Number of night shifts per month	−1.242	0.376	−0.094	−3.303	***P** **=** **0.001** **<** **0.05***
Whether working overtime	0.032	1.18	0.001	0.027	0.979
Whether to participate in AI–related knowledge training	−2.41	0.935	−0.068	−2.577	***P** **=** **0.01** **<** **0.05***
AI literacy	0.523	0.073	0.214	7.142	*** < 0.001***
Perceived organizational support	1.729	0.074	0.629	23.326	*** < 0.001***
Self–efficacy	0.527	0.166	0.069	3.171	***P** **=** **0.002** **<** **0.05***

R^2^ = 0.794, adjustR^2^ = 0.789; F = 156.816, *P* < 0.001. Bold values indicate statistical significance (*P* < 0.05).

## Discussion

### Digital resilience scores of clinical nurses

The moderate-to-high level of digital resilience observed in this cohort suggests that clinical nurses possess a foundational capacity to cope with digital transformation (9). However, the relatively low score in the Perceiving Digital Risks dimension indicates a critical weakness: nurses may be less aware of potential digital threats such as data security breaches, patient privacy violations, and over-reliance on technology (34). This finding aligns with previous reports that digital training for nurses has predominantly focused on operational skills rather than risk awareness (35).

In fast-paced clinical environments, nurses often focus primarily on completing diagnostic and therapeutic tasks. This leaves little room for critical reflection on latent safety threats. Such threats may arise from system flaws or improper use. Examples include alarm fatigue or misjudgment due to information overload (36). Therefore, nursing administrators should prioritize enhancing risk perception as a core component of digital resilience training (37).

The moderate score in the Learning Knowledge and Skills dimension further reflects the challenge of rapid technological iteration. Pre-service digital knowledge quickly becomes obsolete. Heavy clinical workloads also hinder nurses from updating their competencies. A lack of systematic continuing digital education adds to this challenge. As a result, nurses cannot keep pace with technological advancement (38).

Given the uneven performance across dimensions, nursing managers should move beyond generalized “IT skills” training and establish a tiered, targeted development system for digital resilience (39). Priority should be given to integrating digital security and ethical decision-making into routine training through workshops and case studies (40). Additionally, creating a sustained micro-learning resource library and encouraging digital innovation practices based on clinical scenarios can improve nurses' ability to adapt to technological updates (24). Although the Overcoming Digital Stress dimension scored relatively higher, its absolute level still indicates room for improvement. Healthcare institutions should pay attention to mental wellbeing in digital work environments. They can promote digital wellness strategies, such as rational allocation of digital tasks and advocating for “digital disconnect” periods. These strategies will help foster a sustainable digital work ecosystem for nurses (41).

### Factors influencing digital resilience

#### Monthly income

Monthly income level was a significant correlate of digital resilience. Higher-income nurses demonstrated greater resilience. This association may be indirectly related to the accessibility of personal resources and career development stages reflected by income (42). Higher income often enables nurses to acquire better electronic devices. It also allows access to more efficient learning platforms or fee-based advanced digital skill training. These resources help nurses accumulate a more solid foundational competency (43).

Income level is often correlated with professional title, position, and years of service. Nurses with higher incomes are typically at mid-career stages or in key clinical roles. They have accumulated richer clinical experience and adaptive capabilities, including systems thinking and psychological adjustment skills. A cross-sectional survey of pediatric nurses found that resilience correlates positively with age, work experience, professional title, education level, and monthly income (44). Similarly, a latent class analysis of 805 nurses revealed that monthly income is an influential factor in nurse resilience patterns. Psychological rigidity was significantly associated with monthly income below RMB 5,000 (45).

Managers should consider establishing special grants for digital skills enhancement. They should also provide in-hospital free digital training resources and optimize performance incentives to recognize improvements in digital capability. These measures can help mitigate competency gaps potentially exacerbated by economic disparities (46).

#### Night shift frequency

Nurses with fewer night shifts per month exhibited higher levels of digital resilience. The underlying mechanisms may include: (1) stable daily routines and adequate recovery time, which help preserve cognitive clarity and emotional stability when facing digital learning tasks (47); (2) frequent night shifts leading to cumulative fatigue, decreased attention, and emotional exhaustion, thereby depleting the psychological resources necessary for handling digital challenges such as technical complexity and information overload (48); and (3) more regular work schedules providing greater opportunities to participate in on-the-job training and team-based learning, facilitating systematic improvement of digital literacy (49). These findings highlight the importance of scientifically managing human resources. Hospitals should optimize shift schedules and reduce unnecessary night shift frequency. They should also provide targeted digital skill support and psychological resources for nurses who work nights (50).

#### AI-related training and AI literacy

Consistent with the multivariate regression result (β = −2.410, *P* = 0.010), nurses who had participated in AI training demonstrated higher digital resilience than those who had not. This finding supports the value of AI training in enhancing digital resilience. Notably, a multi-center study of 901 nurses in China reported that only 14.24% had received AI training, with significant regional disparities (Beijing outperforming Yunnan) (16).

Higher AI literacy was a significant factor associated with greater digital resilience, consistent with the consensus in nursing education and technology empowerment (37). Zeng et al. (16) surveyed 901 nurses across three Chinese provinces. They found that AI literacy promotes nurses' AI usage intention both directly (30.51%) and indirectly. The indirect effects were mediated by AI self-efficacy (21.41%) and general attitudes (14.58%). Self-efficacy played a particularly crucial mediating role.

Nurses with higher AI literacy can move beyond technical operation. They can deeply understand the fundamental principles of intelligent systems. They also grasp the underlying data logic and potential limitations in clinical decision-making (51). The N.U.R.S.E.S. framework provides a structured approach to AI integration, emphasizing that nurses need to recognize AI's benefits (e.g., enhanced decision-making) and challenges (e.g., biased data), with ethical considerations guiding AI usage (17). These nurses may face human–machine judgment conflicts or system failures. When this happens, they can more accurately identify the cause of the problem. They then select the most appropriate coping strategy. As a result, they avoid experiencing generalized helplessness or frustration (35).

Nursing managers should evolve AI-related training. Training should move from sporadic, voluntary formats to an institutionalized, structured mandatory system. It should also be combined with case-based teaching and practical training grounded in clinical scenarios. A qualitative study explored Chinese nurses‘ perceptions of GAI training. It revealed that nurses' use of GAI to support scientific research is dynamic. Their perceptions and practices evolve over time. Specialized training helps nurses transition from “GAI-dominated” to “nurse-dominated” approaches (52).

#### Perceived organizational support

Perceived organizational support was a key facilitating factor for digital resilience. Building digital resilience requires continuous investment of energy and emotional resources, a process that can easily lead to resource depletion. A scoping review of 66 studies demonstrated the critical role of meso-level organizational environments in fostering and sustaining healthcare workers‘ resilience. These environments include interpersonal dynamics, supervisory support, and psychosocial workplace conditions. The review also challenged the notion that resilience is solely an individual trait (53). Individuals with adequate support systems demonstrate greater resilience. They are better equipped to cope with stress and adversity. This directly helps them overcome digital pressure and adapt to new environments. Social exchange theory further posits that employees perceive whether the organization values and cares for them. When they feel valued, a sense of obligation arises. This obligation is based on the principle of reciprocity. It motivates them to respond with greater adaptability and resilience. A cross-sectional survey was conducted on 111 British nurses during the COVID-19 pandemic. It revealed significant relationships between perceived organizational support and nurses‘ resilience. Lower perceived support was associated with lower resilience levels and higher burnout (54). Managers should consciously integrate the enhancement of perceived organizational support into their overall strategy. This strategy should foster digital resilience through multi-dimensional support. Such support includes institutional frameworks, resources, and emotional backing. A study of 565 medical staff demonstrated that organizational support serves as a chain mediator between digital literacy and work engagement. This finding highlights the importance of organizational support structures in enabling digital competency development (55). Specific measures include the following. First, solidify resource support. This means ensuring digital training and technical assistance are readily available. Second, foster emotional support. Leaders should publicly acknowledge efforts and demonstrate tolerance for mistakes during transitional phases. Third, design supportive systems. These systems should allow sufficient transition periods for digital transformation and incorporate progress into positive performance incentives.

#### Self-efficacy

A significant positive correlation was found between self-efficacy and digital resilience. This finding aligns with previous research (18). Conte et al. surveyed 1,081 Italian nurses and nursing students. They found that only 47.1% demonstrated adequate digital self-efficacy. This finding highlights the importance of strengthening this psychological resource (18).

When individuals face threats in digital and real-world contexts, psychological resources are essential for developing resilience. These resources include positive psychological traits such as self-efficacy (11).

Nurses with higher self-efficacy are more likely to view the rapidly evolving digital environment as an opportunity. They see it as a chance to enhance their professional competence rather than an overwhelming burden. When they encounter new digital systems or complex functions, they tend to seek solutions proactively. They may explore online resources or consult colleagues. Similarly, when faced with digital stressors like system failures or information overload, they are more inclined to use positive coping strategies. They view these situations as manageable challenges, not sources of anxiety (27).

Nursing managers can strengthen nurses' self-efficacy beliefs in several ways. They can provide structured opportunities for success in digital tasks. They can establish supportive peer modeling and mentorship systems (20). They can also offer targeted, process-oriented feedback.

#### Limitations and future directions

This study has several limitations. First, the sample came from only two tertiary hospitals in one city, limiting generalizability. Multi-center studies with cross-regional samples are needed. Second, the cross-sectional design prevents causal inferences. Longitudinal or interventional studies are required. Third, the digital resilience scale was developed for nursing undergraduates and needs validation for clinical nurses, although its internal consistency in our sample was high (Cronbach's α = 0.958). Fourth, the model explained 78.9% of variance, which is high for a psychosocial construct. This may reflect strong theoretical alignment or common method bias from self-report measures. Future research should use objective measures and multi-method designs.

These efforts will provide stronger evidence base for enhancing digital resilience among clinical nurses.

## Data Availability

The original contributions presented in the study are included in the article/supplementary material, further inquiries can be directed to the corresponding author.

## References

[1] Cucci F Marasciulo D Romani M Soldano G Cascio D De NG . The contribution of artificial intelligence in nursing education: a scoping review of the literature. Nurs Rep. (2025) 15:283. doi: 10.3390/nursrep15080283 40863670 PMC12389495

[2] Hashish EA Alsayed S Alqarni BHM Alammari NM Alsulami RO Fiorini J. Nurse managers' perspectives on digital transformation and informatics competencies in e-leadership: a qualitative study. J Nurs Manag. (2025) 2025:8178924. doi: 10.1155/jonm/8178924 40726545 PMC12303638

[3] Rony MKK Parvin MR Ferdousi S. Advancing nursing practice with artificial intelligence: enhancing preparedness for the future. Nurs Open. (2024) 11:10.1002/nop2.2070. doi: 10.1002/nop2.2070 38268252 PMC10733565

[4] Mo Y Zhang H Bai X Lei S Han L. Digital resilience of clinical nurses: a concept analysis. BMC Nurs. (2025) 24:1231. doi: 10.1186/s12912-025-03713-6 41029686 PMC12487026

[5] Zhang Z Zhao S Shao Y Duan X Sun P Chen L . The effect of digital competence on nurses' career sustainability: a cross-sectional study. J Nurs Manag. (2024) 2024:8813704. doi: 10.1155/jonm/8813704 40224846 PMC11925313

[6] Chang L Lee P Dowding D Hsieh C Chou K Lee T . Integrating information and communication technology into nursing practice for resilience: a cross-sectional quantitative study. PLoS ONE. (2025) 20:e0324865. doi: 10.1371/journal.pone.0324865 40465704 PMC12136407

[7] Mutlu S Yilmaz E. Psychological resilience and professional quality of life among surgical nurses: a cross-sectional study. BMC Psychol. (2026) 14:322. doi: 10.1186/s40359-026-04114-3 41645273 PMC12973890

[8] Yu F Chu G Yeh T Fernandez R. Effects of interventions to promote resilience in nurses: a systematic review. Int J Nurs Stud. (2024) 157:104825. doi: 10.1016/j.ijnurstu.2024.104825 38901125

[9] Su Y Yu Z Chen S Chen J Wang L. Exploring digital resilience and its related factors among nursing students in the era of AI. Nurse Educ Pract. (2026) 90:104636. doi: 10.1016/j.nepr.2025.104636 41242097

[10] Alotaibi N Wilson C B Traynor M. Enhancing digital readiness and capability in healthcare: a systematic review of interventions, barriers, and facilitators. BMC Health Serv Res. (2025) 25:500. doi: 10.1186/s12913-025-12663-3 40186200 PMC11969766

[11] Larsson M Ho DM Kirschner M Seifritz E Manoliu A. Digital resilience interventions for healthcare workers: a systematic review. Front Psychiatry. (2025) 16:1519670. doi: 10.3389/fpsyt.2025.1519670 41000340 PMC12457347

[12] Dornan M. Every nurse an AI nurse: a framework for integrating artificial intelligence across nursing practice, education, research and policy. Digit Health. (2025) 11:20552076251377939. doi: 10.1177/20552076251377939 41036436 PMC12480800

[13] Gustavson AM Miller MJ Boening N Hudson EM Wisdom JP Burke RE . Identifying factors influencing emerging innovations in hospital discharge decision making in response to system stress: a qualitative study. BMC Health Serv Res. (2024) 24:1293. doi: 10.1186/s12913-024-11784-5 39468596 PMC11520429

[14] Wishart AV Hamouda RK Elsaady E Aslam MR Perala A Khan S. The adoption and impact of telemedicine on health equity: a narrative review from the Jamaican context. Cureus. (2024) 16:e69650. doi: 10.7759/cureus.69650 39301454 PMC11412623

[15] Lin TC. Mobile nursing information system utilization: the task-technology fit perspective. Comput Inform Nurs. (2014) 32:129–37. doi: 10.1097/CIN.0000000000000039 24419090

[16] Zeng Q Huang X Zhu J Su S Hu Y Zhang X. Mechanisms of nurses' AI use intention formation in Sichuan, Yunnan, and Beijing, China: mediating effects of AI literacy via self-efficacy-to-attitude pathways. Front Public Health. (2025) 13:1622802. doi: 10.3389/fpubh.2025.1622802 40709030 PMC12287104

[17] Hoelscher SH Pugh A. N.U.R.S.E.S embracing artificial intelligence: a guide to artificial intelligence literacy for the nursing profession. Nurs Outlook. (2025) 73:102466. doi: 10.1016/j.outlook.2025.102466 40561583

[18] Conte G Arrigoni C Magon A De AG Paglione G Baroni I . Unlocking the determinants of digital and technological self-efficacy: insights from a cross-sectional study among nurses and nursing students. Healthcare. (2025) 13:129–37. doi: 10.3390/healthcare13172208 40941558 PMC12427988

[19] Di MVE Perego G Milano F Cugnata F Brombin C Catarinella A . Preparedness for the digital transition in healthcare: insights from an Italian sample of professionals. Healthcare. (2025) 13. doi: 10.3390/healthcare13202556 41154235 PMC12563548

[20] Walzer S Armbruster C Mahler S Farin-Glattacker E Kunze C. Factors influencing the implementation and adoption of digital nursing technologies: systematic umbrella review. J Med Internet Res. (2025) 27:e64616. doi: 10.2196/64616 40743516 PMC12355146

[21] Chen Y Ho KY Zong X Weng Y Yuan C Yorke J. The digital intelligent precise nursing framework: theory development in health recommender system. BMC Nurs. (2025) 24:1191. doi: 10.1186/s12912-025-03830-2 41013575 PMC12465332

[22] Williamson GR. Misrepresenting random sampling? a systematic review of research papers in the Journal of advanced nursing. J Adv Nurs. (2003) 44:278–88. doi: 10.1046/j.1365-2648.2003.02803.x 14641398

[23] Green SB. How many subjects does it take to do a regression analysis. Multivariate Behav Res. (1991) 26:499–510. doi: 10.1207/s15327906mbr2603_7 26776715

[24] He T Huang J Li Y Wang L Liu J Zhang F . The mediation effect of AI self-efficacy between AI literacy and learning engagement in college nursing students: a cross-sectional study. Nurse Educ Pract. (2025) 87:104499. doi: 10.1016/j.nepr.2025.104499 40784134

[25] Galura S Warshawsky N Hu W Utt L. A survey of interim nurse managers to understand the role and the impact on nurse and patient outcomes. J Nurs Adm. (2022) 52:42–50. doi: 10.1097/NNA.0000000000001101 34897206

[26] Qin S Zhang J Sun X Meng G Zhuang X Jia Y . A scale for measuring nursing digital application skills: a development and psychometric testing study. BMC Nurs. (2024) 23:366. doi: 10.1186/s12912-024-02030-8 38822276 PMC11140889

[27] Li F Ma Q Yang C Zhong M. Investigating key elements of digital resilience among nursing undergraduates: a qualitative study. Front Med. (2024) 11:1452580. doi: 10.3389/fmed.2024.1452580 PMC11825263 39959609

[28] Luo D Song Y Cai X Li R Bai Y Chen B . Nurse managers' burnout and organizational support: the serial mediating role of leadership and resilience. J Nurs Manag. (2022) 30:4251–61. doi: 10.1111/jonm.13852 36205051

[29] Berdida DJE Lopez V Grande R A N. Nursing students' perceived stress, social support, self-efficacy, resilience, mindfulness and psychological well-being: a structural equation model. Int J Ment Health Nurs. (2023) 32:1390–404. doi: 10.1111/inm.13179 37249199

[30] Pan Q Lan M Tan CY Tao S Liang Q Law N. Development and validation of digital resilience scale for primary and secondary school students. BMC Psychol. (2025) 13:1412. doi: 10.1186/s40359-025-03739-0 41291973 PMC12750681

[31] Hertzog M A. Considerations in determining sample size for pilot studies. Res Nurs Health. (2008) 31:180–91. doi: 10.1002/nur.20247 18183564

[32] Polit D F Beck C T. The content validity index: are you sure you know what's being reported? Critique and recommendations. Res Nurs Health. (2006) 29:489–97. doi: 10.1002/nur.20147 16977646

[33] Yu M Wang H Wu Y Zhang Q Du X Huang X . The influence of emotional intelligence on psychiatric nurses' care behavior, and the chain mediating role of compassion fatigue and perception of management. J Psychosoc Nurs Ment Health Serv. (2025) 63:35–43. doi: 10.3928/02793695-20241101-02 39508679

[34] Atalla ADG El-Gawad MMA Hashish EAA Elseesy NAM Abd EKMAI Sobhi MSM. Embracing artificial intelligence in nursing: exploring the relationship between artificial intelligence-related attitudes, creative self-efficacy, and clinical reasoning competency among nurses. BMC Nurs. (2025) 24:661. doi: 10.1186/s12912-025-03306-3 40542340 PMC12181922

[35] Dzikowicz DJ Aktas M Wang J Dreisbach C. An evaluation of funded national institutes of health grants in nursing science and artificial intelligence. Nurs Outlook. (2025) 73:102529. doi: 10.1016/j.outlook.2025.102529 40803105 PMC12451553

[36] Shaoru C Hui Z Su W Ruxin J Huiyi Z Hongmei Z . Determinants of medical equipment alarm fatigue in practicing nurses: a systematic review. SAGE Open Nurs. (2023) 9:23779608231207227. doi: 10.1177/23779608231207227 37927965 PMC10621293

[37] El-Banna MM Sajid MR Rizvi MR Sami W McNelis AM AI. Literacy and competency in nursing education: preparing students and faculty members for an AI-enabled future-a systematic review and meta-analysis. Front Med. (2025) 12:1681784. doi: 10.3389/fmed.2025.1681784 41384110 PMC12689331

[38] Huang M Y. Empowering medical students with AI literacy: a curriculum development journey. Med Educ. (2025) 59:550–51. doi: 10.1111/medu.15654 40016929

[39] Pilosof NP Welcman Y Barrett M Oborn E Barrett S. Building digital resilience: leading healthcare transformation through an online community. Front Digit Health. (2025) 7:1656804. doi: 10.3389/fdgth.2025.1656804 40978698 PMC12446309

[40] Alzaabi A Masters K. Assessing medical students' readiness for artificial intelligence after pre-clinical training. BMC Med Educ. (2025) 25:824. doi: 10.1186/s12909-025-07008-x 40457325 PMC12131332

[41] Quek FF Meldrum S Hislop J. A systematic scoping review of the current applications of digital technology in undergraduate surgical education. Cureus. (2025) 17:e77278. doi: 10.7759/cureus.77278 39801700 PMC11725316

[42] Chen X Zhu L Gong S He Y Chen H Zhao X. Population characteristics and influencing factors of clinical nurses' informatics competency: a latent profile analysis. West J Nurs Res. (2025) 47:641–50. doi: 10.1177/01939459251333686 40271955

[43] Perimal-Lewis L Correia S V Sakellari E. Editorial: socioeconomic inequalities in digital health. Front Digit Health. (2025) 7:1680350. doi: 10.3389/fdgth.2025.1680350 41451378 PMC12730157

[44] Pu Z Zhou W Zhao J Bian L. Psychological resilience in pediatric nurses: a cross-sectional survey. Transl Pediatr. (2025) 14:2748–57. doi: 10.21037/tp-2025-390 41216455 PMC12597174

[45] Zhao S Zhang Z Duan X Shao Y Wang F Chen Y . Resilience and flexibility for clinical nurses: a latent class analysis. J Nurs Manag. (2024) 2024:6171305. doi: 10.1155/2024/6171305 40224750 PMC11918684

[46] Neter E Western MJ Cooper R Silva AM König LM. Towards bridging the digital divide: training healthcare professionals for digitally inclusive healthcare systems. Glob Health Res Policy. (2025) 10:31. doi: 10.1186/s41256-025-00433-x 40739280 PMC12309028

[47] Xie J Ma R Guo C Li Y Liu K Cheng L. The effects of the night shift on cognitive function and cerebral oxygenation/hemodynamics in sub-plateau-area PICU nurses. Sci Rep. (2025) 15:25365. doi: 10.1038/s41598-025-10158-z 40659723 PMC12260080

[48] Qtait M Alia M F Jaradat Y. The impact of rotating shift work on nurse burnout: a systematic review of contributing factors and organizational strategies. SAGE Open Nurs. (2025) 11:23779608251374232. doi: 10.1177/23779608251374232 40919288 PMC12409028

[49] Mayes P Schott-Baer D. Professional development for night shift nurses. J Contin Educ Nurs. (2010) 41:17–22. doi: 10.3928/00220124-20091222-05 20102138

[50] Kang HW Kim J Kim KJ Bae EK Kang H Jang JH . Shift nurses' work quality and job satisfaction after implementing the Inha University hospital nursing AI scheduling system (IH-NASS). BMC Nurs. (2025) 24:792. doi: 10.1186/s12912-025-03470-6 40597282 PMC12210576

[51] Si J. Exploring AI literacy, attitudes toward AI, and intentions to use AI in clinical contexts among healthcare students in Korea: a cross-sectional study. BMC Med Educ. (2025) 25:1233. doi: 10.1186/s12909-025-07766-8 40885994 PMC12398176

[52] Tong L Niu Y Zhou L Jin S Wang Y Xiao Q. Perceptions and experiences of generative artificial intelligence training to support research for Chinese nurses: a qualitative focus group study. Int J Nurs Sci. (2025) 12:210–7. doi: 10.1016/j.ijnss.2025.04.010 40529465 PMC12168451

[53] Oprea N Giacomelli G Sartirana M Trinchero E Georgescu I. Meso-organisational determinants of healthcare workers' resilience: results of a scoping review. Health Policy. (2025) 161:105412. doi: 10.1016/j.healthpol.2025.105412 40912178

[54] Abdulmohdi N. The relationships between nurses' resilience, burnout, perceived organisational support and social support during the second wave of the COVID-19 pandemic: a quantitative cross-sectional survey. Nurs Open. (2024) 11:e2036. doi: 10.1002/nop2.2036 38268251 PMC10697858

[55] Wu J Zhang Q Gan X Liu X Hu J Wang Y. The relationship between digital literacy and innovative behavior among Chinese medical students: the chain mediating roles of diversity experience and creative self-efficacy. BMC Med Educ. (2025) 25:1194. doi: 10.1186/s12909-025-07799-z 40849469 PMC12374281

